# PEBP1 and 15-LO-1 in Asthma: Biomarker Potential for Diagnosis and Severity Stratification

**DOI:** 10.3390/diagnostics15111322

**Published:** 2025-05-24

**Authors:** Vijayalakshmi Vadde, Mohammed Kaleem Ullah, Mandya Venkateshmurthy Greeshma, Muhlisa Muhammed Ali Laila, Athira Nair, Sivasubramaniam Karunakaran, SubbaRao V. Madhunapantula, Sindaghatta Krishnarao Chaya, Komarla Sundararaja Lokesh, Jayaraj Biligere Siddaiah, Padukudru Anand Mahesh

**Affiliations:** 1 Department of Respiratory Medicine, JSS Medical College, JSS Academy of Higher Education & Research (JSS AHER), Mysuru 570015, India; vijjichowdary6@gmail.com (V.V.); greeshmamv@jssuni.edu.in (M.V.G.); muhlisamr@gmail.com (M.M.A.L.); athiranair94@gmail.com (A.N.); sivasubramaniam1810@gmail.com (S.K.); chaya.sindaghatta@gmail.com (S.K.C.); lokeshpulmo@gmail.com (K.S.L.); bsjayaraj@jssuni.edu.in (J.B.S.); 2 Center of Excellence in Molecular Biology and Regenerative Medicine (CEMR) Laboratory (DST-FIST Supported Center and ICMR Collaborating Center of Excellence—ICMR-CCoE), Department of Biochemistry (DST-FIST Supported Department), JSS Medical College, JSS Academy of Higher Education & Research (JSS AHER), Mysuru 570015, India; ka7eem@gmail.com (M.K.U.); mvsstsubbarao@jssuni.edu.in (S.V.M.); 3 Division of Infectious Disease and Vaccinology, School of Public Health, University of California, Berkeley, CA 94720, USA

**Keywords:** ferroptosis, PEBP1, 15-LO-1, PEBP1/15-LO-1, asthma, asthma severity

## Abstract

**Background:** Ferroptosis, a regulated form of cell death characterized by iron-dependent lipid peroxidation, has been implicated in the pathogenesis of asthma. The ferroptosis markers PEBP1 and 15-LO-1 are increasingly recognized as potential biomarkers for asthma. This study investigates the association of these markers with asthma and its severity to evaluate their diagnostic potential. **Methods:** This cross-sectional study included 45 asthmatic patients and 45 healthy controls. Serum phosphatidylethanolamine-binding protein 1 (PEBP1) and 15-lipoxygenase-1 (15-LO-1) levels were measured using ELISA. Spirometric parameters (FEV_1_, FEV_1_/FVC ratio, and PEFR) were recorded. A multivariate regression assessed associations between ferroptosis markers and asthma severity. A generalized linear model (GLM) analyzed the relationship between biomarkers (PEBP1 and 15-LO-1) and lung function parameters. A receiver operating characteristic (ROC) analysis evaluated the discriminative capacity of PEBP1 and 15-LO-1. **Results:** PEBP1 and 15-LO-1 levels were significantly associated with asthma. The multivariate analysis revealed that low PEBP1 levels were strongly associated with asthma and severe asthma (*p* < 0.001). While elevated 15-LO-1 levels were associated with asthma (*p* < 0.001), they did not correlate with severity. The ROC analysis demonstrated excellent discriminative capacity for PEBP1 (AUC 0.962, cutoff 1509.8 pg/mL) and 15-LO-1 (AUC 0.895, cutoff 144.8 pg/mL). Lower PEBP1 and higher 15-LO-1 levels were associated with reduced lung function, and lower FEV_1_, FEV_1_/FVC, and PEF. Older age and female gender were associated with severe asthma. **Conclusions:** PEBP1 and 15-LO-1 are promising biomarkers for asthma, with PEBP1 showing strong correlations with asthma severity. These findings highlight the potential role of ferroptosis markers in asthma and underscore the need for further longitudinal studies to explore these markers’ clinical utility in personalized asthma management.

## 1. Introduction

Asthma, a prevalent chronic respiratory disease, poses a significant global health burden due to its complexity and nature [1,2]. Characterized by symptoms such as wheezing, shortness of breath, chest tightness, and coughing, asthma exhibits variations over time and intensity, compounded by expiratory airflow limitation. The disease’s impact is unevenly distributed across regions and socioeconomic groups, as shown in studies like the Indian study on the epidemiology of asthma (INSEARCH), which highlights its considerable national burden and underscores its global significance [3]. Recent insights from the Global Burden of Disease (GBD) report emphasize the urgent need for innovative strategies for understanding and managing asthma [4,5]. Alarmingly, disparities in asthma-related mortality and disability-adjusted life years (DALYs) in India compared to global averages underscore the necessity for improved therapeutic strategies and disease management [6].

**Figure 1:**
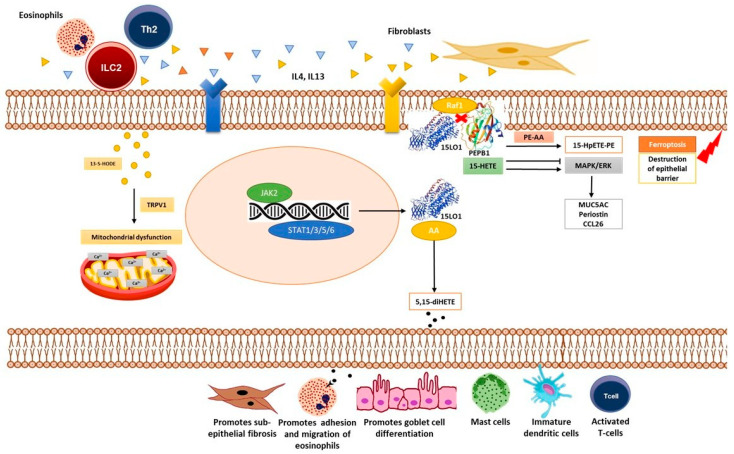
Mechanistic pathway illustrating how IL-4 and IL-13 activate the JAK2/STAT and Raf1/15-LO-1 pathways in asthma, leading to ferroptosis through PEBP1/15-LO-1 activation. This process contributes to epithelial barrier destruction, eosinophil migration, goblet cell differentiation, and sub-epithelial fibrosis, worsening asthma pathology. Note: Th2: T helper 2 cells; ILC2s: group 2 innate lymphoid cells; IL-4: Interleukin-4; IL-13: Interleukin-13; JAK2: Janus Kinase 2; STAT: Signal Transducer and Activator of Transcription; 13-S-HODE: 13-S-hydroxyoctadecadienoic acid; TRPV1: Transient Receptor Potential Vanilloid 1; Raf1: Rapidly Accelerated Fibrosarcoma 1; 15-LO-1: 15-Lipoxygenase-1; PEBP1: Phosphatidylethanolamine-Binding Protein 1; 5,15-diHETE: 5,15-dihydroxy-eicosatetraenoic acid; 15-HpETE-PE: 15-hydroperoxy-eicosatetraenoic acid-phosphatidylethanolamine; MAPK: Mitogen-Activated Protein Kinase; ERK: Extracellular Signal-Regulated Kinase; MUC5AC: Mucin 5AC; and CCL26: C-C Motif Chemokine Ligand 26.

The exploration of novel biomarkers that can aid in the precise diagnosis, risk stratification, and therapeutic targeting of asthma is needed. Ferroptosis, a type of regulated cell death, identified in 2012, has emerged as a promising biomarker, which is distinctly mediated by iron dependency and lipid peroxidation [7]. The phosphatidylethanolamine-binding protein 1 (PEBP1)/15-lipoxygenase (15-LO-1) complex, a key mediator of ferroptosis, is found to be elevated in asthma patients [8]. This elevation contributes to airway redox imbalance and triggers T2 inflammation, thereby exacerbating asthma symptoms [9]. Ferroptosis is fundamentally driven by the iron-dependent accumulation of lipid reactive oxygen species (ROS) and the consequential lipid peroxidation [10]. This process is intricately regulated by various enzymes and molecules, including Glutathione Peroxidase 4 (GPX4) and the PEBP1/15-LO-1 complex [10]. The role of ferroptosis in asthma, particularly through the PEBP1/15-LO-1 axis, has been implicated in linking lipid peroxidation to inflammatory pathways, offering insights into the disease’s underlying mechanisms [7]. In asthmatic conditions, interleukin-4 (IL-4) and interleukin-13 (IL-13) are predominantly produced by T helper 2 (Th2) cells, group 2 innate lymphoid cells (ILC2s), and eosinophils [11,12,13]. These cytokines activate the Janus Kinase 2/Signal Transducer and Activator of Transcription 1/3/5/6 (JAK2/STAT1/3/5/6) pathway [14], contributing to mitochondrial dysfunction via 13-S-hydroxy octadecadienoic acid (13-S-HODE) and Transient Receptor Potential Vanilloid 1 (TRPV1) activation [15,16]. Simultaneously, IL-4 and IL-13 stimulate fibroblasts [17], leading to the activation of the Rapidly Accelerated Fibrosarcoma 1 (Raf1) and 15-Lipoxygenase-1 (15-LO-1) pathways [18]. This results in the production of 5,15-dihydroxy-eicosatetraenoic acid (5,15-diHETE) and 15-hydroperoxy-eicosatetraenoic acid-phosphatidylethanolamine (15-HpETE-PE) [18], which subsequently activates the Mitogen-Activated Protein Kinase/Extracellular Signal-Regulated Kinase (MAPK/ERK) pathway [17]. This cascade promotes the expression of Mucin 5AC (MUC5AC), Periostin, and C-C Motif Chemokine Ligand 26 (CCL26), contributing to epithelial barrier destruction via ferroptosis, sub-epithelial fibrosis, eosinophil migration, goblet cell differentiation, and the activation of mast cells, immature dendritic cells, and T-cells hallmark features of asthma [19,20,21,22,23] (Figure 1).

PEBP1 and 15-LO-1 are integral to lipid peroxidation and play a central role in the ferroptotic pathway [20]. Their activity is further influenced by complex interactions with cytokine signaling, particularly the Th2 cytokine IL-13, which promotes the formation of hydroperoxy phospholipids and exacerbates cellular redox imbalance [20,24]. Given the intricate relationship between ferroptosis and asthma, there is a compelling need for a detailed comparative study of ferroptosis markers, specifically PEBP1 and 15-LO-1 [10]. There are limited clinical studies on these ferroptosis markers in asthma patients [8,20,24,25,26,27]. We hypothesize that the dysregulation of serum ferroptosis markers, specifically an imbalance in PEBP1 and 15-LO-1 levels, is associated not only with the presence of asthma but also with its severity. We propose that these markers, individually or as a ratio, hold potential as accessible, non-invasive biomarkers for asthma phenotyping and severity stratification. This study aims to evaluate the clinical utility of serum PEBP1 and 15-LO-1 levels, and their ratio, as potential biomarkers of asthma, and explores their association with disease severity and lung function impairment.

## 2. Materials and Methods

### 2.1. Study Design and Population

A cross-sectional case–control [hospital-based (cases) and community-based (controls)] observational study was conducted at the Department of Respiratory Medicine, JSS Medical College and Hospital, Mysore, Karnataka. This study included 90 participants (45 asthma patients and 45 healthy controls) and was carried out between November 2022 and June 2024. This study received approval from the Institutional Ethics Committee (approval number: JSS/MC/PG/IEC-23/2022-23).

### 2.2. Inclusion and Exclusion Criteria

- The inclusion criteria were as follows:
  - Confirmed asthmatics aged 18–65 years, diagnosed according to the Global Initiative for Asthma (GINA) 2022 guidelines [1].
- The exclusion criteria were as follows:
  - Patients with conditions like cancer, renal diseases, hepatic diseases, and neurogenerative disorders;
  - Patients aged below 18 years or above 65 years;
  - Pregnant individuals;
  - Patients who did not provide informed consent.

### 2.3. Recruitment and Diagnostic Criteria

Participants were recruited from the outpatient and inpatient departments of the JSS Hospital, South India. Asthma was diagnosed and severity was classified into moderate and severe asthma based on GINA guidelines [1]. Spirometry was performed adhering to the American Thoracic Society (ATS) standards [28]. A detailed demographic and clinical questionnaire followed by pre- and post-bronchodilator spirometry was performed, and clinical and sociodemographic information of study subjects was collected which included age, gender, BMI, Smoking history, and detailed medical history. The National Asthma Education and Prevention Program (NAEPP) classification was used, which relies on assessing the severity of asthma such as asthma symptoms and lung function at the time the patient is being evaluated and prior to the commencement of treatment. Three variables are considered in classifying asthma severity, namely, daytime symptoms, nighttime symptoms, and lung function [29].

### 2.4. Control Group

The control group consisted of healthy individuals with no history of respiratory diseases or symptoms and normal spirometry. Controls were selected from the community in the same geographic area as the asthma cases and were age- and gender-matched.

### 2.5. Sample Collection and Processing

Venous blood samples (5 mL) were collected from each participant under aseptic conditions. The samples were allowed to clot for 30 min and then centrifuged at 3000 rpm for 10 min within 2 h of collection of blood to isolate serum. The separated serum was stored in Eppendorf tubes at −80 degrees Celsius for further analysis. Hematological parameters were analyzed using an automated blood analyzer Sysmex XN 1000 (Sysmex Corp., Kobe, Japan), and the data were recorded. NLR (Absolute neutrophil count/Absolute lymphocyte count), PLR (Absolute platelet count/Absolute lymphocyte count), and AEC (Absolute Eosinophil Count) were calculated.

### 2.6. Enzyme-Linked Immunosorbent Assay

Serum levels of PEBP1 and 15-LO-1 were measured using commercially available enzyme-linked immunosorbent assay (ELISA) kits: Human PEBP1 (Catalogue No: BZEK2350) and Human 15-LO-1 (Catalogue No: BZEK2351) from Chongqing Biospes Co., Ltd., Chongqing, China. The assays were performed according to the manufacturer’s instructions using a standard sandwich ELISA format. The detection range for PEBP1 was 37.5–600 pg/mL, with a sensitivity of 3 pg/mL, while for 15-LO-1, the detection range was 3–48 pg/mL, with a sensitivity of 0.3 pg/mL. All the serum samples were diluted at a ratio of 1:5 with the kit’s recommended diluent prior to analysis to ensure optimal measurement accuracy. Final concentrations were calculated by multiplying the raw values by the dilution factor. Therefore, reported concentrations may appear higher than the assay’s stated detection range, but they remain valid due to this dilution adjustment. Absorbance was recorded at 450 nm using the iMark™ Microplate Absorbance Reader (Bio-Rad, Inc., Hercules, CA, USA).

### 2.7. Statistical Analysis

Categorical variables were expressed as percentages, while continuous variables were presented as the mean ± standard deviation (SD) or median and interquartile range (IQR). Comparative analyses were conducted using chi-square tests for categorical variables and independent *t*-tests or one-way ANOVA for continuous variables. Receiver operating characteristic (ROC) curves were generated to evaluate the diagnostic utility of PEBP1 and 15-LO-1 levels. Multivariate logistic regression was used to determine the association between PEBP1 and 15-LO-1 between various factors. A generalized linear model (GLM) was used to assess the relationship between PEBP1 and lung function parameters, as well as Eosinophil % and AEC. Similarly, the association between 15-LO-1 and these parameters was analyzed using GLM. Additionally, GLM was performed to evaluate the association between the 15-LO-1/PEBP1 ratio and lung function parameters, along with Eosinophil % and AEC. The data were log-transformed to calculate the 15-LO-1/PEBP1 ratio, ensuring normalization and minimizing skewness before conducting all subsequent analyses. All the statistical analyses were conducted using jamovi-2.3.28 (The jamovi Project, Sydney, Australia) software. Two-tailed tests with a significance threshold of *p* < 0.05 were applied.

## 3. Results

The demographic and clinical characteristics of asthmatic and non-asthmatic participants are summarized in Table 1. No significant differences were observed in age, gender distribution, BMI, or smoking status between the two groups. However, asthmatics had a significantly higher prevalence family history of asthma (*p* < 0.001) compared to non-asthmatics. While FVC pre percent predicted did not differ significantly (*p* = 0.17), asthmatics showed significantly lower FEV_1_ pre percent predicted (*p* < 0.001), pre FEV_1_/FVC ratios (*p* < 0.001), and PEF pre % predicted (*p* < 0.001). In terms of biomarkers, PEBP1 levels were significantly lower in asthmatics (*p* < 0.001), while 15-LO-1 levels and the 15-LO-1/PEBP1 ratio were significantly higher (*p* < 0.001) in asthmatics.

The demographic and clinical characteristics of moderate and severe asthmatics compared to non-asthmatic controls are presented in Table 2. Age showed a significant difference across the groups, with severe asthmatics being older than both moderate asthmatics and controls (*p* < 0.001). There were no significant differences in gender distribution or BMI among the groups. Although smoking prevalence did not reach statistical significance (*p* = 0.19), a higher percentage of severe asthmatics (23.5%) were smokers compared to moderate asthmatics (7.1%) and controls (8.9%). Allergy prevalence was significantly higher in asthmatic groups (*p* < 0.001), with the most common triggers being dust in moderate asthmatics (50.0%) and severe asthmatics (70.6%). Family history of asthma was significantly more common in moderate asthmatics (75.0%) compared to severe asthmatics (41.2%) and absent in controls (*p* < 0.001). Lung function parameters, including FVC, FEV_1_, FEV_1_/FVC ratio, FEF 25–75%, and PEF, were significantly reduced in asthmatics, particularly in the severe group (*p* < 0.001). The biomarker analysis revealed significantly lower PEBP1 levels and higher 15-LO-1 levels and 15-LO-1/PEBP1 ratio in both moderate and severe asthmatics compared to controls (*p* < 0.001).

**Figure 2:**
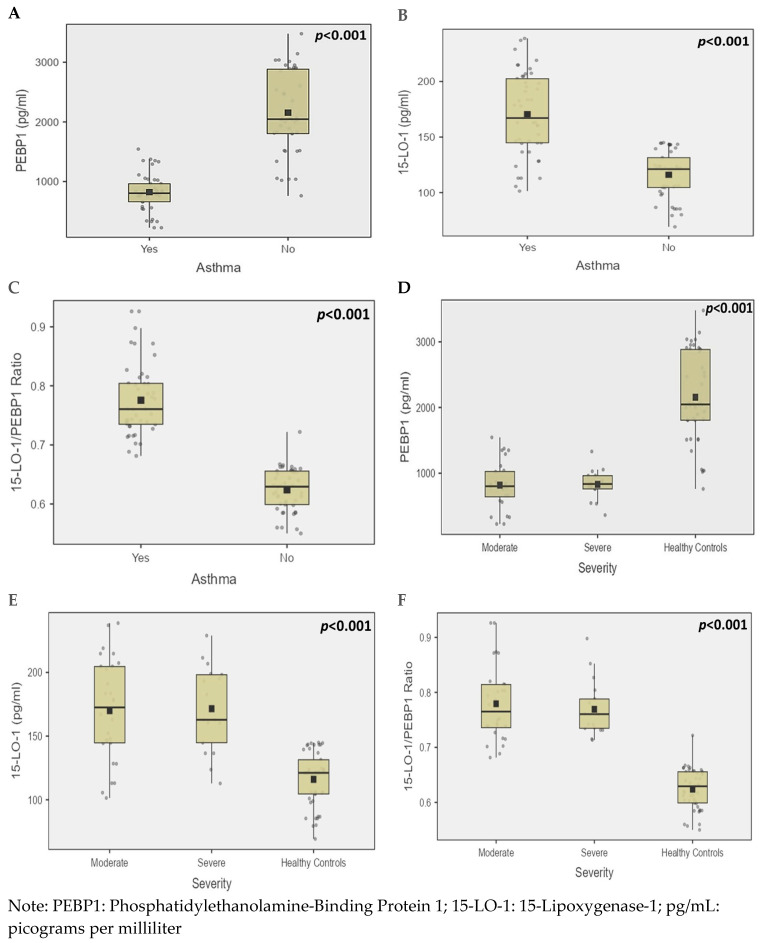
The serum concentrations of PEBP1 (pg/mL), 15-LO-1 (pg/mL) and 15-LO-1/PEBP1 ratio across different asthma severity groups. (**A**–**C**) displays PEBP1 (pg/mL), 15-LO-1 (pg/mL) and 15-LO-1/PEBP1 ratio levels, respectively, in individuals with and without asthma, while (**D**–**F**) illustrate PEBP1 (pg/mL), 15-LO-1 (pg/mL), and 15-LO-1/PEBP1 ratio levels, respectively, categorized by asthma severity (moderate, severe, and healthy controls).

The box plots (Figure 2) illustrate the distribution of PEBP1 (pg/mL), 15-LO-1 (pg/mL) and 15-LO-1/PEBP1 ratio serum concentrations across different groups. Figure 2A–C compare individuals with and without asthma, showing significantly reduced PEBP1 (pg/mL), elevated 15-LO-1 (pg/mL) and 15-LO-1/PEBP1 ratio levels in the asthma group. Figure 2D–F further stratify the data by asthma severity (moderate, severe, and healthy controls), revealing a decrease in PEBP1 (pg/mL), and increase in 15-LO-1 (pg/mL) and 15-LO-1/PEBP1 ratio levels.

**Figure 3:**
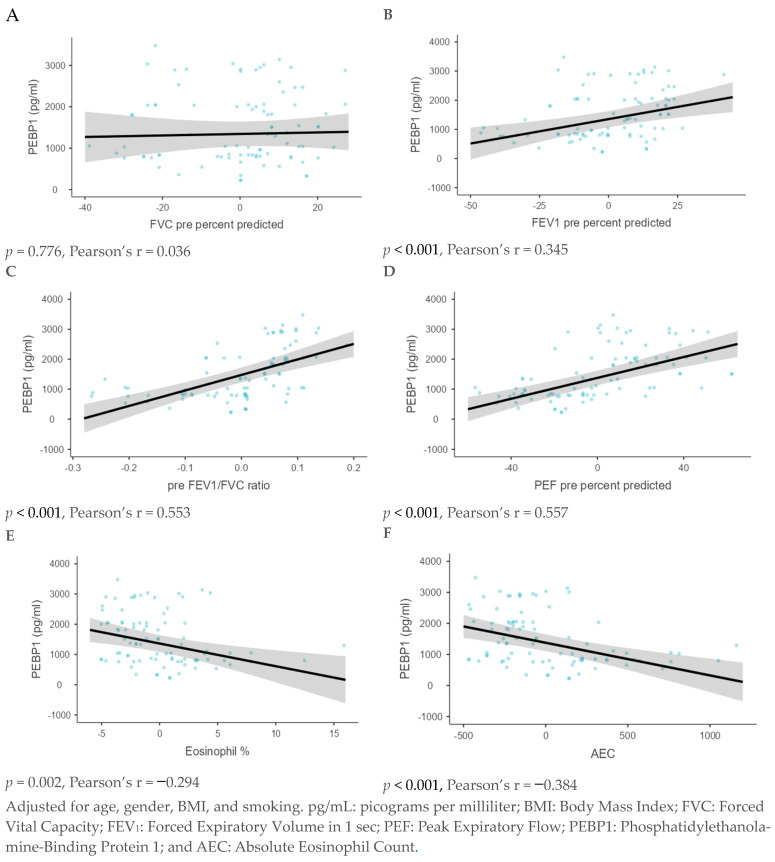
A generalized linear model analysis was done to analyze the association between PEBP1 and the lung function parameters, including the percentage of predicted FVC (**A**), percentage of predicted FEV_1_ (**B**), pre-FEV_1_/FVC ratio (**C**), and pre-percent predicted PEF (**D**), Eosinophil % (**E**), and AEC (**F**).

Using the generalized linear model analysis, a significant positive association was observed between PEBP1 levels and FEV_1_ pre-percent predicted, pre-FEV_1_/FVC ratio, and PEF pre-percent predicted (*p* < 0.001 for each). In contrast, Eosinophil %, and AEC showed a significant negative association (*p* < 0.001 for each). However, no significant association was observed with FVC pre-percent predicted (Figure 3).

**Figure 4:**
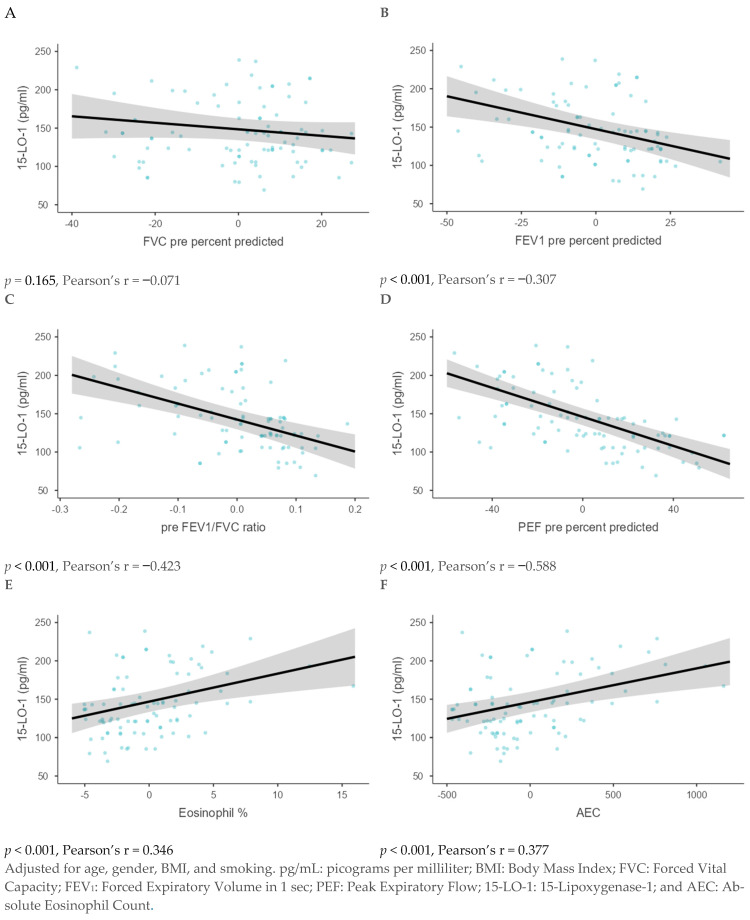
A generalized linear model analysis was performed to analyze the association between 15-LO-1 and the lung function parameters, including the percentage of predicted FVC (**A**), percentage of predicted FEV_1_ (**B**), pre-FEV_1_/FVC ratio (**C**), and pre-percent predicted PEF (**D**), Eosinophil % (**E**), and AEC (**F**).

Using the GLM analysis, 15-LO-1 levels demonstrated a significant negative association with FEV_1_ pre-percent predicted, pre-FEV_1_/FVC ratio, and PEF pre-percent predicted (*p* < 0.001 for each). In contrast, Eosinophil %, and AEC showed a significant positive association (*p* < 0.001 for each). However, no significant association was observed with FVC pre-percent predicted (Figure 4).

**Figure 5:**
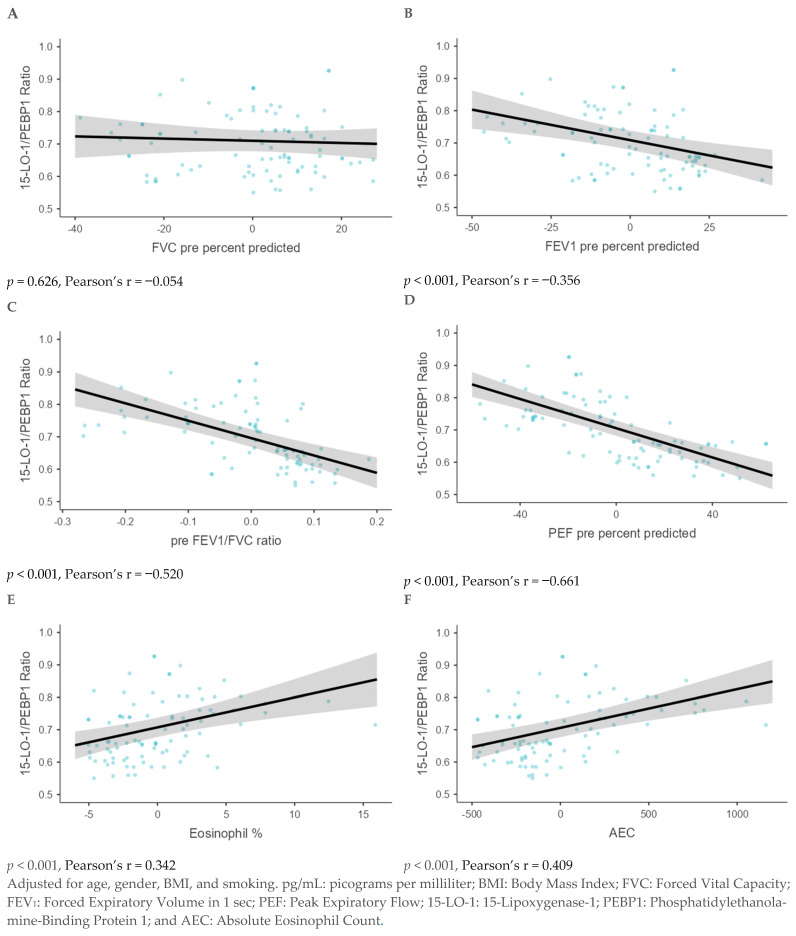
A generalized linear model analysis was performed to assess the association between the 15-LO-1/PEBP1 ratio and lung function parameters, including the percentage of predicted FVC (**A**), percentage of predicted FEV_1_ (**B**), pre-FEV_1_/FVC ratio (**C**), pre-percent predicted PEF (**D**), Eosinophil % (**E**), and AEC (**F**).

Using GLM analysis, a significant association was observed between the 15-LO-1/PEBP1 ratio and multiple parameters, including FEV_1_ pre-percent predicted, pre-FEV_1_/FVC ratio, and PEF pre-percent predicted (*p* < 0.001 for each). In contrast, Eosinophil %, and AEC showed a significant positive association (*p* < 0.001 for each). However, no significant association was observed with FVC pre-percent predicted (Figure 5).

A similar analysis was carried with only asthma cases, and we observed no significant association between 15-LO-1, PEBP1, and 15-LO-1/PEBP1 ratio and FVC pre-percent predicted, FEV_1_ pre-percent predicted, pre-FEV_1_/FVC ratio, PEF pre-percent predicted, Eosinophil %, and AEC (Supplementary Figures S1A–F–S3A–F). However, in healthy controls, no association was found with these parameters (Supplementary Figures S4A–F, S5A–C,E,F and S6A–F). A notable exception was a significant negative association between 15-LO-1 and PEF pre-percent predicted (Figure S5D) (*p* = 0.004)

**Figure 6:**
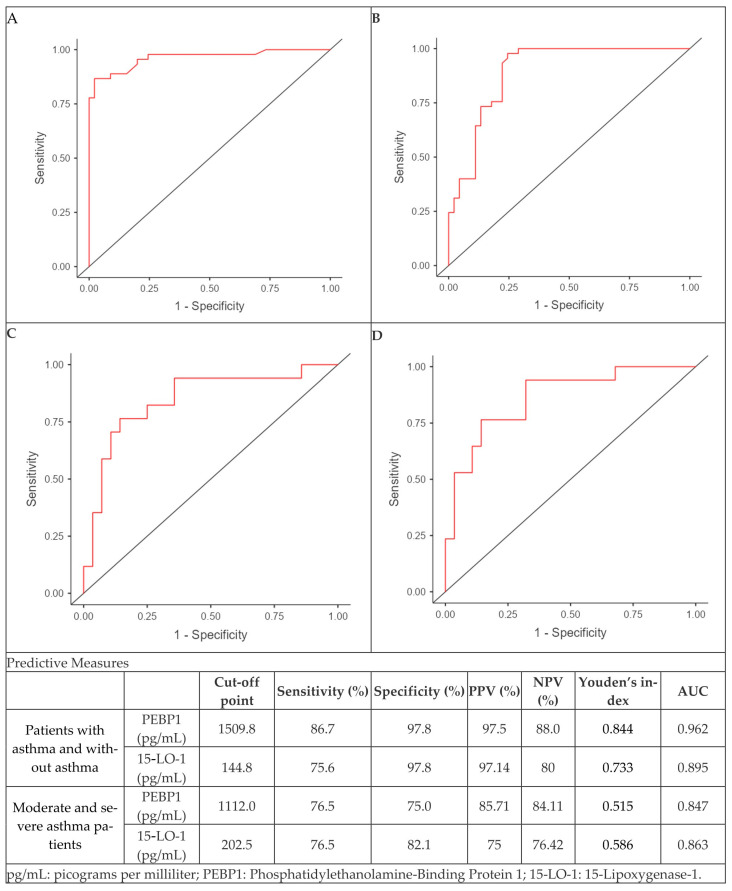
ROC curve for quantifying PEBP1 in asthmatic and non-asthmatic participants (**A**), and in moderate and severe asthmatics (**B**), ROC curve for quantifying 15-LO-1 in asthmatic and non-asthmatic participant (**C**), and in moderate and severe asthmatics (**D**).

The ROC curves indicate that both PEBP1 and 15-LO-1 exhibit strong predictive capability to discriminate between asthmatic and non-asthmatic participants. Notably, PEBP1 showed superior sensitivity (86.7%) and specificity (97.8%) at the optimal cutpoint of 1509.8 pg/mL, yielding an AUC of 0.962. Similarly, 15-LO-1 demonstrated strong discriminative ability at a cut-off of 144.8 pg/mL, with a sensitivity of 75.6%, specificity of 97.8%, and an AUC of 0.895. Similarly, in the context of moderate and severe asthma, PEBP1 and 15-LO-1 demonstrated predictive performance, with AUCs of 0.847 and 0.863, respectively. Youden’s index values indicate that PEBP1 and 15-LO-1 are particularly effective in discriminating between subjects with asthma and without asthma (Figure 6).

In the multivariate analysis, PEBP1 and 15-LO-1 levels were significantly associated with asthma. Patients with PEBP1 levels < 1509.8 pg/mL had markedly higher odds of asthma (OR = 416.71, 95% CI: 58.33–11,043.30, *p* < 0.001). Similarly, patients with 15-LO-1 levels ≥144.8 pg/mL had significantly elevated odds of asthma (OR = 262.54, 95% CI: 37.57–6178.39, *p* < 0.001). These findings suggest that reduced PEBP1 and elevated 15-LO-1 levels are strongly associated with asthma (Table 3).

In the multivariate analysis, age, gender, BMI, and PEBP1 levels were significantly associated with asthma severity. Older age was associated with severe asthma across all models (*p* < 0.05). Female gender showed a higher likelihood of severe asthma, with significantly elevated odds in all models (*p* < 0.05). Patients with PEBP1 levels <1112.0 pg/mL had a significantly higher risk of severe asthma in Model 1 (OR = 216.15, 95% CI: 1.92–215,555.09, and *p* < 0.05) and Model 3 (OR = 227.57, 95% CI: 2.03–210,000.33, and *p* < 0.05). These findings indicate that older age, female gender, and lower PEBP1 levels are key predictors of severe asthma (Table 4).

## 4. Discussion

This cross-sectional study investigates the relationship between ferroptosis markers, specifically PEBP1, 15-LO-1, and 15-LO-1/PEBP1 ratio, and their association with asthma and its severity. Ferroptosis, a regulated form of cell death characterized by iron-dependent lipid peroxidation, has emerged as a critical player in various diseases, including asthma [30,31]. Our findings from the multivariate analysis demonstrate that low PEBP1 and high 15-LO-1 are significantly and independently associated with asthma, but only low PEBP1 is associated with severe asthma; similarly, the 15-LO-1/PEBP1 ratio showed a significant association with asthma. The strength of association for 15-LO-1 alone and the 15-LO-1/PEBP1 ratio were similar. ROC analyses demonstrated the exceptional ability of PEBP1 and 15-LO-1 to distinguish asthmatic patients from healthy controls, with AUC values of 0.962 and 0.895, respectively. Additionally, GLM analyses, adjusted for age, gender, BMI, and smoking, revealed a strong correlation between these markers and spirometric parameters (lower FEV_1_, lower FEV_1_/FVC ratio, and lower PEFR), eosinophil % and AEC, indicative of disease severity. These findings represent a significant step in understanding ferroptosis markers in asthma and suggest potential therapeutic targets for future research.

This study revealed significant age differences among patients with moderate asthma, and severe asthma (*p* < 0.01), with severe asthma patients being older on average. This aligns with findings by Zhao et al. [8] who described an age-related imbalance in cell survival and death mechanisms in patients with asthma. Interestingly, while gender differences in asthma severity were not statistically significant in this study (*p* = 0.44), similar conclusions were reached by Fuseini et al. [32] and Yamada et al. [33], who found no significant gender-specific differences in ferroptosis-related pathways or asthma severity. These results suggest that both male and female patients are equally affected by the molecular processes driving asthma, although subtle differences warrant further investigation.

Asthma remains a complex, heterogeneous condition characterized by airway inflammation and hyperresponsiveness [34]. While spirometry is the diagnostic gold standard, it often fails to capture the full spectrum of disease, particularly in patients who do not meet spirometric criteria yet continue to experience symptoms and exacerbations [35]. This diagnostic gap emphasizes the need for reliable biomarkers that reflect the diverse clinical manifestations and molecular pathways involved in asthma. The concept of asthma endotypes, which categorize disease subsets based on distinct biological mechanisms, has gained prominence in recent years, emphasizing the importance of molecular markers for personalized management strategies [36,37].

Recent research implicates ferroptosis in asthma pathogenesis [38,39,40], with the PEBP1/15-LO-1 complex playing a central role. The increased colocalization of PEBP1 and 15-LO-1 in the airway epithelial cells of asthmatic patients [20] suggests that ferroptosis actively contributes to airway dysfunction [8]. Most of the current research available in the literature has focused on elucidating the role of PEBP1 and 15-LO-1 in the pathogenesis of asthma [8,20,24,25,26,27]. However, their association with the severity of asthma and interplay with various other contributory risk factors and correlation with clinical and laboratory parameters have not been extensively studied, which makes the present study one of the very few that have delved into this aspect of the research in the field.

When compared to other studies, such as those by Xu et al. [24] and Nagasaki et al. [9], our findings support the notion that oxidative stress, driven by pathways involving 15-LO-1, plays a crucial role in asthma severity. Specifically, Xu and colleagues discussed how increased oxidative stress and disrupted cellular homeostasis contribute to ferroptosis and potentially worsen asthma outcomes. Similarly, Nagasaki’s [9] study emphasized the role of oxidative imbalance in severe asthma, particularly the involvement of T2-associated proteins, which may correlate with our observation of increased 15-LO-1 levels in more severe cases. In asthma, several cytokines such as IL-4, IL-5, IL-13, and VEGF play key roles in disease pathogenesis by promoting airway eosinophilia, mucus overproduction, bronchial hyperresponsiveness, immunoglobulin synthesis, and angiogenesis [41]. Additionally, 15-LO-1 has been reported to inhibit VEGF, suggesting it may modulate the angiogenic process in asthma [42]. The GLM plots from our study demonstrate an inverse relationship between asthma severity and PEBP1 levels, and a direct relationship between severity and 15-LO-1 levels, though the correlation strength appears moderate. Interestingly, the lack of correlation between 15-LO-1 and lung function parameters in the asthma-only analysis (as shown in the Supplementary Data) could be due to the immune heterogeneity of asthma. Since 15-LO-1 expression is predominantly associated with the T2 signature, its role in airway obstruction may be more pronounced in T2-high asthma, whereas severe non-T2 asthma can also exhibit significant lung function decline [22,43]. This may explain why a positive correlation between 15-LO-1 and lung function parameters emerges only when healthy controls who have normal spirometry and likely no T2 inflammation are included in the analysis. This is consistent with findings by Zhao et al. [8], who identified a balance between PEBP1 and 15-LO-1 as a key factor in determining cell survival in the asthmatic epithelium. Similarly, Chen et al. [26] highlighted the clinical importance of these biomarkers in guiding the development of targeted therapies, reinforcing the broader relevance of our findings within the field of asthma research. In summary, our study contributes to the growing body of evidence that suggests a significant link between ferroptosis markers and asthma severity, highlighting the potential of PEBP1 and 15-LO-1 as biomarkers for assessing and managing asthma.

Future research should focus on elucidating the specific mechanisms through which ferroptosis impacts asthma severity and explore targeted interventions that can modulate these pathways to improve clinical outcomes for asthmatic patients. By understanding the intricate interplay between ferroptosis markers and asthma, we can develop more effective, tailored therapeutic strategies that enhance both patient care and quality of life.

### Strength and Limitations

This study has several limitations, including a small sample size, which may have reduced the statistical power. The cross-sectional design limits causal inferences, and unmeasured confounders such as environmental factors or genetics may have influenced results. The lack of longitudinal data restricts insights into asthma progression and the long-term effects of ferroptosis markers. Additionally, potential bias in self-reported data could affect the outcomes. The focus on specific ferroptosis markers may overlook other relevant pathways, and the single-center design limits the generalizability of the findings. Furthermore, our study did not include measurements of 15-LO-1 activity markers, such as 15-HETE, 12-HETE, or hydroperoxyl derivatives, which are important for confirming the functional implications of increased 15-LO-1 in ferroptosis. Further research with larger cohorts and longitudinal data is needed to better understand the causal mechanisms and clinical utility of PEBP1 and 15-LO-1.

## 5. Conclusions

In conclusion, our study underscores the pivotal role of ferroptosis markers, lung function parameters, and quality of life in the management and prognosis of asthma severity. The significant correlations between lower PEBP1 levels, higher 15-LO-1 levels, and severe asthma highlight the potential of these markers as therapeutic targets.

## Figures and Tables

**Table 1:** Demographic and clinical characteristics of asthmatic vs. non-asthmatics.

	Asthmatics (*N* = 45)	Healthy Controls (*N* = 45)	*p*-Value
Age in years	45.0 (24.0–55.0)	43.0 (32.7–51.3)	0.89 *
Gender			0.20 ^†^
Male	22.0 (48.9%)	28.0 (62.2%)	
Female	23.0 (51.1%)	17.0 (37.8%)	
BMI (kg/m^2^)	22.9 (21.6–28.0)	24.3 (22.5–25.9)	0.68 *
Smoking			0.50 ^†^
No	39.0 (86.7%)	41.0 (91.1%)	
Yes	6.0 (13.3%)	4.0 (8.9%)	
Allergy triggers			
Dust	26.0 (57.8%)	0.0 (0.0%)	
Fumes and smoke	2.0 (4.4%)	0.0 (0.0%)	
No identified triggers	9.0 (20.0%)	45.0 (100%)	
Seasonal symptoms	8.0 (17.8%)	0.0 (0.0%)	
Family history			<0.001 ^†^
No	17.0 (37.8%)	45.0 (100.0%)	
Yes	28.0 (62.2%)	0.0 (0.0%)	
PFT parameters			
FVC pre-percent predicted	84.0 (68.0–90.3)	88.0 (75.3–95.0)	0.17 *
FEV_1_ pre-percent predicted	68.0 (56.0–80.3)	86.0 (72.3–95.0)	<0.001 *
Pre FEV_1_/FVC ratio	0.7 (0.6–0.8)	0.8 (0.8–0.8)	<0.001 *
PEF pre % predicted	58.0 (43.0–71.0)	96.0 (86.7–113.3)	<0.001 *
Levels of biomarkers			
PEBP1 (pg/mL)	805.2 (662–984.0)	2046.1 (1805.2–2884.8)	<0.001 *
15-LO-1 (pg/mL)	167.1 (144.7–203.2)	121.1 (103.4–133.3)	<0.001 *
15-LO-1/PEBP1 ratio	0.76 (0.73–0.80)	0.62 (0.59–0.65)	<0.001 *
Eosinophil %	6.7 (3.3–8.6)	3.0 (1.6–5.0)	<0.001 *
AEC	640.0 (246.7–863.3)	300.0 (233.3–400.0)	<0.001 *

* Mann–Whitney U test. ^†^ Pearson’s Chi-squared test. Continuous variables are presented as median (IQR), while categorical variables are expressed as *n* (%); BMI: Body Mass Index; FVC: Forced Vital Capacity; FEV_1_: Forced Expiratory Volume in 1 s; PEF: Peak Expiratory Flow; PEBP1: Phosphatidylethanolamine-Binding Protein 1; 15-LO-1: 15-Lipoxygenase-1; pg/mL: picograms per milliliter; and AEC: Absolute Eosinophil Count.

**Table 2:** Demographic and clinical characteristics of moderate and severe asthmatic vs. non-asthmatics.

	Moderate (*N* = 28)	Severe (*N* = 17)	Healthy Controls (*N* = 45)	*p*-Value
Age in years	27.0 (23.0–50.8)	51.0 (45.7–60.0)	43.0 (32.7–51.3)	<0.001 ^#^
Gender				0.44 ^†^
Female	14.0 (50.0%)	9.0 (52.9%)	17.0 (37.8%)	
Male	14.0 (50.0%)	8.0 (47.1%)	28.0 (62.2%)	
BMI (kg/m^2^)	22.5 (20.5–26.2)	26.1 (22.1–29.2)	24.3 (22.5–25.9)	0.16 ^#^
Smoking				0.19 ^†^
No	26.0 (92.9%)	13.0 (76.5%)	41.0 (91.1%)	
Yes	2.0 (7.1%)	4.0 (23.5%)	4.0 (8.9%)	
Duration of the disease	7.8 (5.2–10.6)	10.0 (6.0–14.7)	-	0.039 *
Allergy triggers				<0.001 ^†^
Dust	14.0 (50.0%)	12.0 (70.6%)	4.0 (8.9%)	
Fumes and smoke	2.0 (7.1%)	0.0 (0.0%)	0.0 (0.0%)	
No identified triggers	5.0 (17.9%)	4.0 (23.5%)	41.0 (91.1%)	
Seasonal symptoms	7.0 (25.0%)	1.0 (5.9%)	0.0 (0.0%)	<0.001 ^†^
Family history				<0.001 ^†^
No	7.0 (25.0%)	10.0 (58.8%)	45.0 (100.0%)	
Yes	21.0 (75.0%)	7.0 (41.2%)	0.0 (0.0%)	
PFT parameters				
FVC pre-percent predicted	88.5 (82.8–94.6)	61.0 (55.3–77.0)	88.0 (75.3–95.0)	<0.001 ^#^
FEV_1_ pre-percent predicted	74.0 (66.7–82.0)	49.0 (38.3–60.7)	86.0 (72.3–95.0)	<0.001 ^#^
Pre FEV_1_/FVC ratio	0.7 (0.7–0.8)	0.6 (0.5–0.7)	0.8 (0.8–0.8)	<0.001 ^#^
FEF 25–75 pre % predicted	40.0 (33.0–55.2)	20.0 (13.0–33.3)	-	<0.001 *
PEF pre % predicted	64.5 (57.4–71.0)	43.0 (37.3–49.7)	96.0 (86.7–113.3)	<0.001 ^#^
Levels of biomarkers				
PEBP1 (pg/mL)	803.0 (614.3–1034.0)	837.0 (762.0–964.3)	2046.1 (1805.2–2884.8)	<0.001 ^#^
15-LO-1 (pg/mL)	172.5 (144.6–204.7)	162.8 (142.1–198.5)	121.1 (103.4–133.3)	<0.001 ^#^
15-LO-1/PEBP1 Ratio	0.76 (0.73–0.81)	0.76 (0.73–0.78)	0.62 (0.59–0.65)	<0.001 ^#^
Eosinophil %	6.0 (3.8–8.4)	7.0 (2.7–9.3)	3.0 (1.6–5.0)	<0.001 ^#^
AEC	620.0 (295.8–850.0)	700.0 (180.0–1096.7)	300.0 (233.3–400.0)	<0.001 ^#^

^#^ Kruskal–Wallis. ^†^ Pearson’s Chi-squared test. * Mann–Whitney test. Continuous variables are presented as median (IQR), while categorical variables are expressed as *n* (%); BMI: Body Mass Index; FVC: Forced Vital Capacity; FEV_1_: Forced Expiratory Volume in 1 s; FEF: Forced Expiratory Flow Between 25% and 75% of Vital Capacity; PEF: Peak Expiratory Flow; PEBP1: Phosphatidylethanolamine-Binding Protein 1; 15-LO-1: 15-Lipoxygenase-1; pg/mL: picograms per milliliter; and AEC: Absolute Eosinophil Count.

**Table 3:** Multivariate analysis of demographic and clinical variables to identify independent predictors of asthma.

Dependent: Asthma		No	Yes	OR (Multivariable)	
				Model 1	Model 2
Age in years	Mean (SD)	41.2 (16.0)	41.6 (11.7)	1.00 (0.94–1.06)	0.97 (0.92–1.03)
BMI (kg/m^2^)	Mean (SD)	24.8 (5.4)	24.1 (2.8)	1.03 (0.86–1.22)	0.90 (0.74–1.05)
Smoking	No	39 (48.8)	41 (51.2)	-	-
	Yes	6 (60.0)	4 (40.0)	0.29 (0.01–4.04)	1.29 (0.16–12.73)
Gender	Female	23 (57.5)	17 (42.5)	-	-
	Male	22 (44.0)	28 (56.0)	2.99 (0.58–22.57)	3.63 (0.94–16.59)
PEBP1 (pg/mL)	≥1509.8	44 (88.0)	6 (12.0)	-	-
	<1509.8	1 (2.5)	39 (97.5)	416.71 (58.33–11,043.30) ***	
15-LO-1 (pg/mL)	<144.8	34 (97.1)	1 (2.9)		
	≥144.8	11 (20.0)	44 (80.0)		262.54 (37.57–6178.39) ***

*** = *p* < 0.001. Note: Model 1: PEBP1; Model 2: 15-LO-1. pg/mL: picograms per milliliter; BMI: Body Mass Index; PEBP1: Phosphatidylethanolamine-Binding Protein 1; and 15-LO-1: 15-Lipoxygenase-1.

**Table 4:** Multivariate analysis of demographic and clinical variables to identify independent predictors of severe asthma.

Dependent: Severity		Moderate	Severe	OR (Multivariable)		
				Model 1	Model 2	Model 3
Age in years	Mean (SD)	35.1 (15.8)	51.1 (10.4)	1.10 (1.02–1.21) *	1.09 (1.02–1.18) *	1.09 (1.01–1.21) *
Gender	Male	14 (63.6)	8 (36.4)	-	-	-
	Female	14 (60.9)	9 (39.1)	80.49 (2.95–15,153.93) *	14.26 (1.55–308.71) *	77.30 (3.05–13,683.44) *
BMI (kg/m^2^)	Mean (SD)	23.9 (5.1)	26.3 (5.7)	1.36 (1.08–1.92) *	1.21 (1.01–1.51)	1.36 (1.08–1.92) *
Smoking	No	26 (66.7)	13 (33.3)	-	-	-
	Yes	2 (33.3)	4 (66.7)	44.88 (0.68–10,061.31)	9.47 (0.38–404.05)	39.38 (0.71–9471.83)
Duration of the disease	Mean (SD)	8.0 (3.7)	10.9 (5.7)	1.14 (0.88–1.59)	1.10 (0.87–1.45)	1.14 (0.88–1.60)
AEC	Mean (SD)	619.6 (353.1)	682.4 (475.8)	1.00 (1.00–1.01)	1.00 (1.00–1.01)	1.00 (1.00–1.01)
PLR	Mean (SD)	188.5 (281.9)	134.6 (69.3)	0.98 (0.97–1.00) *	0.99 (0.97–1.00)	0.98 (0.96–1.00) *
NLR	Mean (SD)	2.6 (2.8)	2.6 (1.3)	4.18 (1.09–21.58) *	3.03 (0.84–13.35)	4.45 (1.11–26.59)
PEBP1 (pg/mL)	≥1112.0	6 (85.7)	1 (14.3)	-	-	-
	<1112.0	22 (57.9)	16 (42.1)	216.15 (1.92–215,555.09) *	-	227.57 (2.03–210,000.33) *
15-LO-1 (pg/mL)	<202.5	19 (57.6)	14 (42.4)	-	-	-
	≥202.5	9 (75.0)	3 (25.0)	-	0.88 (0.08–9.46)	0.62 (0.04–10.06)

* = *p* < 0.05,. Note: Model 1 consists of PEBP1; Model 2 consists of 15-LO-1; and Model 3 consists of PEBP1 and 15-LO-1. pg/mL: picograms per milliliter; BMI: Body Mass Index; AEC: Absolute Eosinophil Count; PLR: Platelet–Lymphocytes Ratio; NLR: Neutrophil–Lymphocytes Ratio; PEBP1: Phosphatidylethanolamine-Binding Protein 1; and 15-LO-1: 15-Lipoxygenase-1.

## Data Availability

All data generated or analyzed during this study are included in this published article and are available from the corresponding author upon reasonable request.

## Supplementary Materials

The following supporting information can be downloaded at https://www.mdpi.com/article/10.3390/diagnostics15111322/s1: Figure S1: A generalized linear model analysis was done in asthma patients to analyze the association between PEBP1 (pg/mL) and lung function parameters [percentage of predicted FVC (A), percentage of predicted FEV_1_ (B), pre-FEV_1_/FVC ratio (C), and pre-percent predicted PEF (D)], Eosinophil % (E), and AEC (F) Figure S2: A generalized linear model analysis was done in asthma patients to analyze the association between 15-LO-1 (pg/mL) and lung function parameters [percentage of predicted FVC (A), percentage of predicted FEV_1_ (B), pre-FEV_1_/FVC ratio (C), and pre-percent predicted PEF (D)], Eosinophil % (E), and AEC (F); Figure S3: A generalized linear model analysis was done in asthma patients to analyze the association between 15-LO-1/PEBP1 ratio and lung function parameters [percentage of predicted FVC (A), percentage of predicted FEV_1_ (B), pre-FEV_1_/FVC ratio (C), and pre-percent predicted PEF (D)], Eosinophil % (E), and AEC (F); Figure S4: A generalized linear model analysis was done in healthy controls to analyze the association between PEBP1 (pg/mL) and lung function parameters [percentage of predicted FVC (A), percentage of predicted FEV_1_ (B), pre-FEV_1_/FVC ratio (C), and pre-percent predicted PEF (D)], Eosinophil % (E), and AEC (F); Figure S5: A generalized linear model analysis was done in asthma patients to analyze the association between 15-LO-1 (pg/mL) and lung function parameters [percentage of predicted FVC (A), percentage of predicted FEV_1_ (B), pre-FEV_1_/FVC ratio (C), and pre-percent predicted PEF (D)], Eosinophil % (E), and AEC (F); Figure S6: A generalized linear model analysis was done in asthma patients to analyze the association between 15-LO-1/PEBP1 ratio and lung function parameters [percentage of predicted FVC (A), percentage of predicted FEV_1_ (B), pre-FEV_1_/FVC ratio (C), and pre-percent predicted PEF (D)], Eosinophil % (E), and AEC (F).
