# Supplementary material for: PEBP1 and 15-LO-1 in Asthma: Biomarker Potential for Diagnosis and Severity Stratification
Source: Diagnostics (Basel). 2025 May 24;15(11):1322. doi: 10.3390/diagnostics15111322 (PMC12154271; doi:10.3390/diagnostics15111322)
Supplement: Supplementary file 1 [file diagnostics-15-01322-s001.zip › diagnostics-3517579-supplementary.pdf]

diagnostics

Article

Supplementary: PEBP1 and 15-LO-1 in Asthma: Biomarker Potential for Diagnosis and Severity Stratification

Using GLM analysis in asthma patients, no significant association was observed between PEBP1 (pg/mL) levels and FVC pre-percent predicted, FEV_1 pre-percent predicted, pre-FEV_1/FVC ratio, PEF pre-percent predicted, Eosinophil % and AEC (Figure S1). Similarly, 15-LO-1 (pg/mL) levels (Figure S2) and 15-LO-1/PEBP1 ratio (Figure S3) showed no significant association with these parameters.

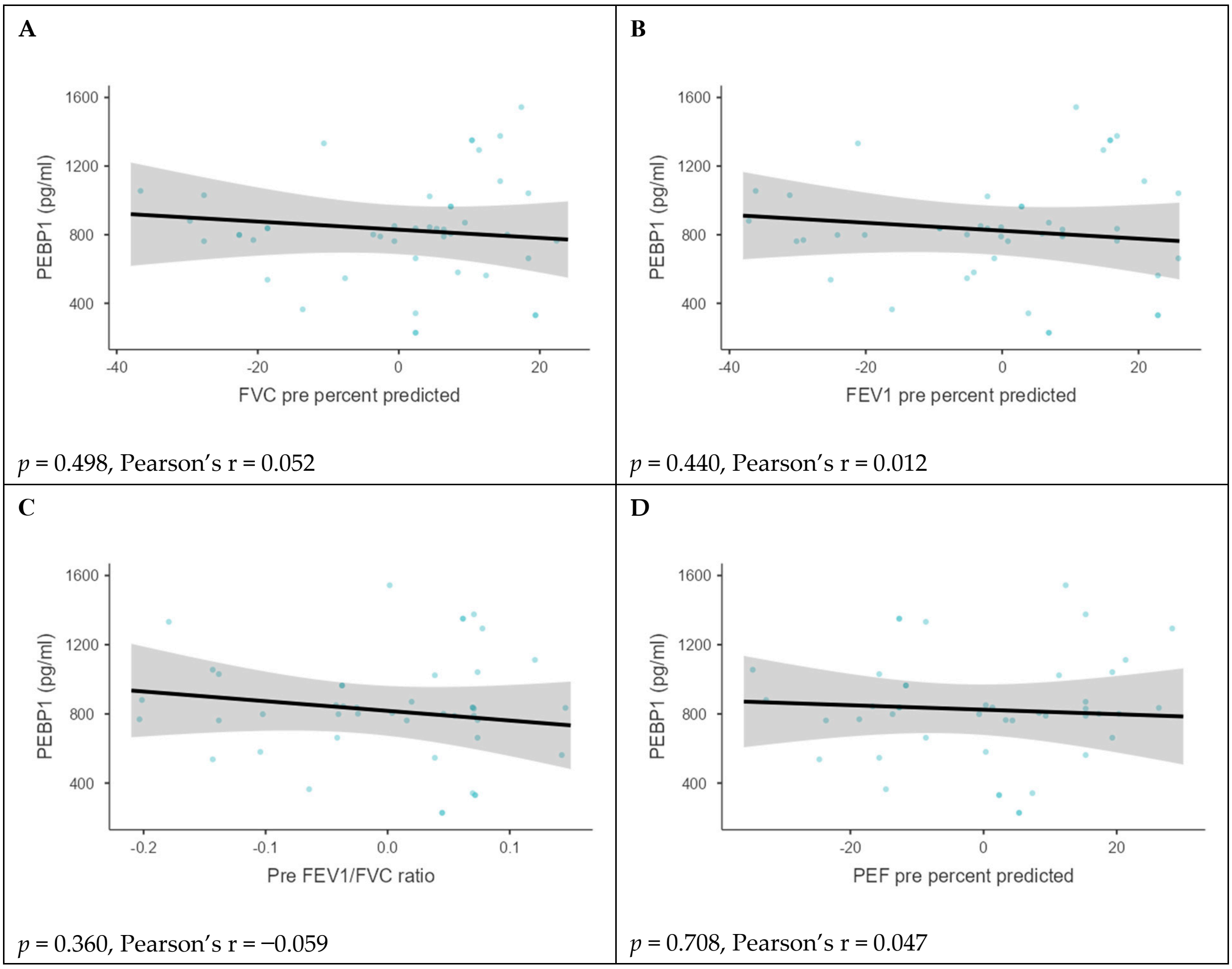

A: $p = 0.498$, Pearson's r = 0.052

B: $p = 0.440$, Pearson's r = 0.012

C: $p = 0.360$, Pearson's r = −0.059

D: $p = 0.708$, Pearson's r = 0.047

E

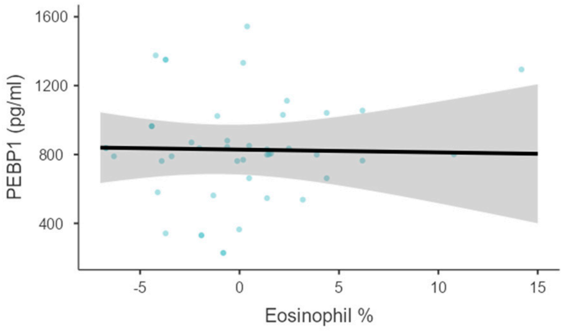

p = 0.891, Pearson's r = 0.109

F

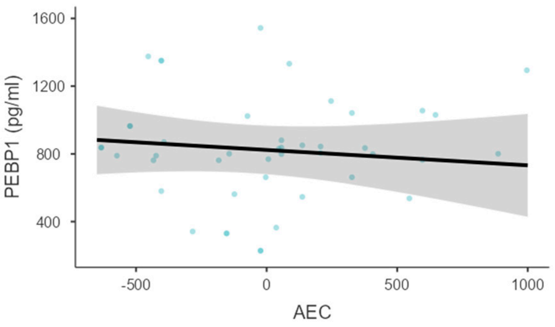

p = 0.465, Pearson's r = 0.044

Note: Analysis done for asthma patients only. Adjusted for Age, Gender, BMI and Smoking. pg/mL: picograms per milliliter; BMI: Body Mass Index; FVC: Forced Vital Capacity; FEV_1: Forced Expiratory Volume in 1 sec; PEF: Peak Expiratory Flow; PEBP1: Phosphatidylethanolamine-Binding Protein 1; AEC: Absolute Eosinophil Count.

Figure S1: A generalized linear model analysis was done in asthma patients to analyze the association between PEBP1 (pg/mL) and lung function parameters [percentage of predicted FVC (**A**), percentage of predicted FEV_1 (**B**), pre-FEV_1/FVC ratio (**C**), and pre-percent predicted PEF (**D**)], Eosinophil % (**E**), and AEC (**F**)].

A

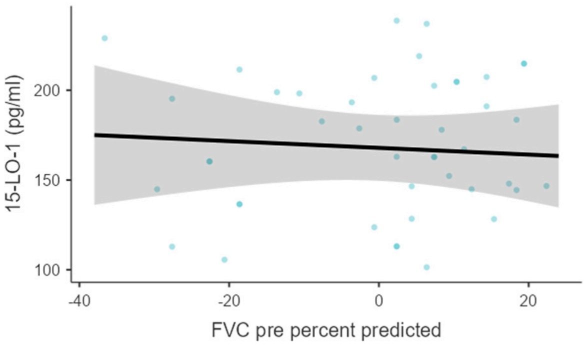

p = 0.678, Pearson's r = 0.075

B

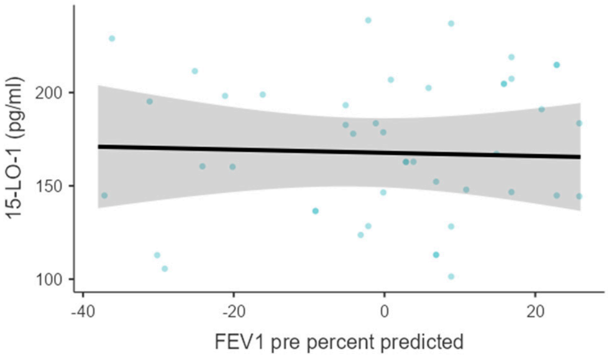

p = 0.824, Pearson's r = 0.096

C

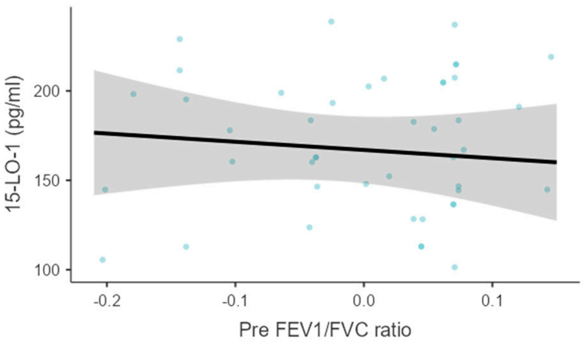

p = 0.559, Pearson's r = 0.042

D

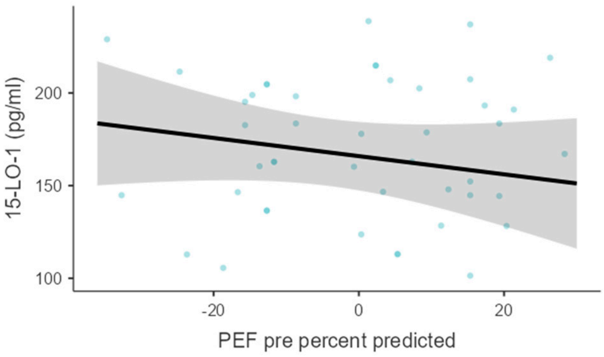

p = 0.268, Pearson's r = −0.008

E

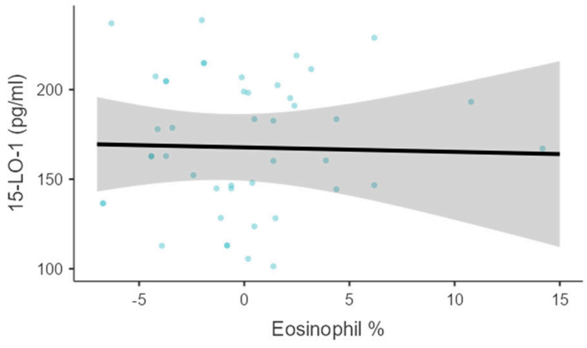

p = 0.870, Pearson's r = 0.052

F

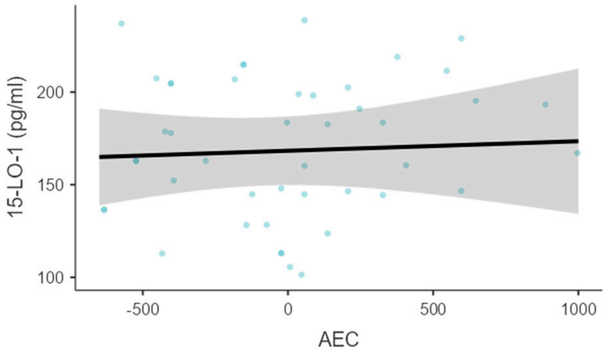

p = 0.748, Pearson's r = 0.123

Note: Analysis done for asthma patients only. Adjusted for Age, Gender, BMI and Smoking. pg/mL: picograms per milliliter; BMI: Body Mass Index; FVC: Forced Vital Capacity; FEV_1: Forced Expiratory Volume in 1 sec; PEF: Peak Expiratory Flow; 15-LO-1: 15-Lipoxygenase-1; AEC: Absolute Eosinophil Count.

Figure S2: A generalized linear model analysis was done in asthma patients to analyze the association between 15-LO-1 (pg/mL) and lung function parameters [percentage of predicted FVC (**A**), percentage of predicted FEV_1 (**B**), pre-FEV_1/FVC ratio (**C**), and pre-percent predicted PEF (**D**)], Eosinophil % (**E**), and AEC (**F**)].

A

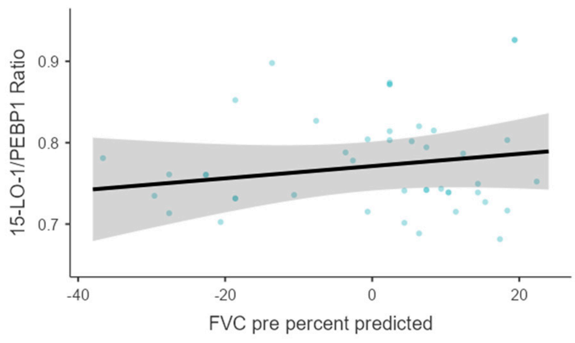

p = 0.312, Pearson's r = 0.093

B

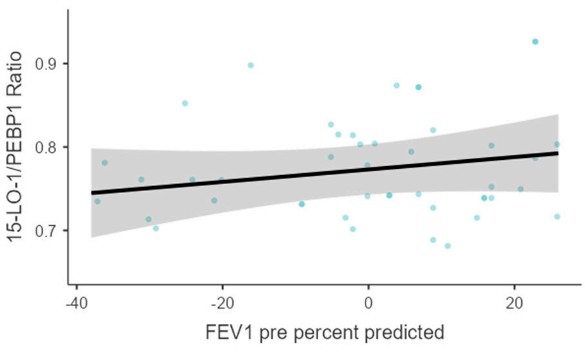

p = 0.240, Pearson's r = 0.140

C

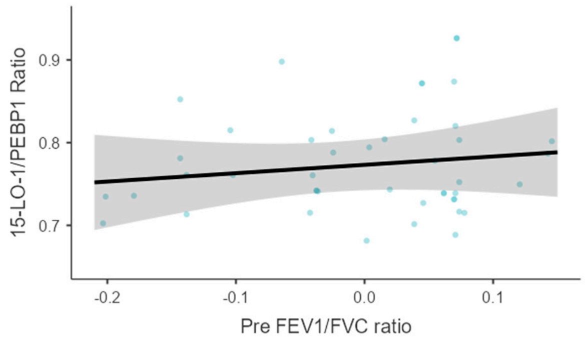

p = 0.436, Pearson's r = 0.138

D

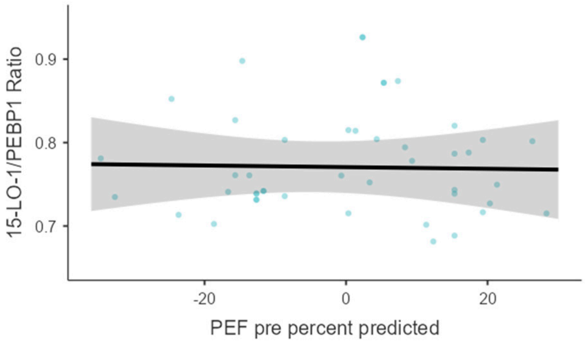

p = 0.893, Pearson's r = −0.008

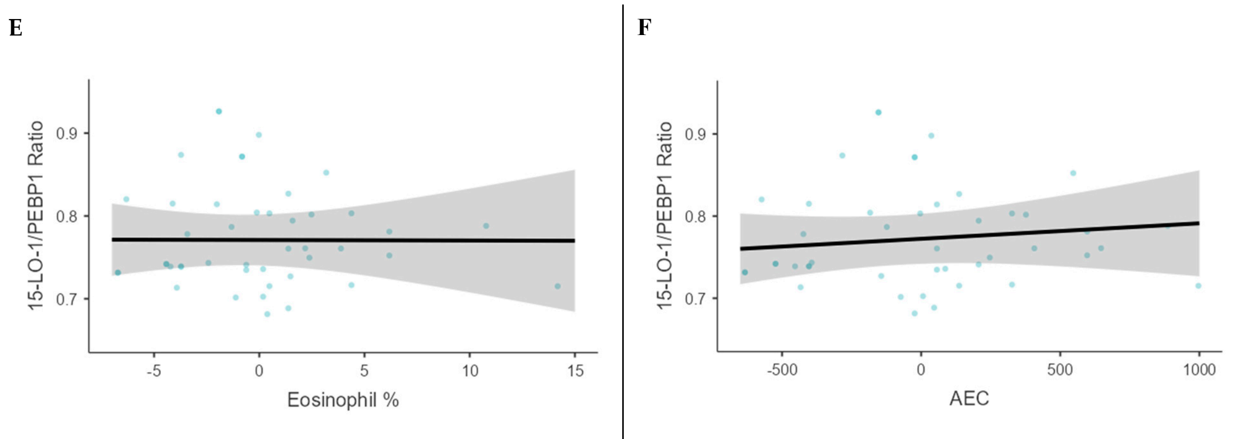

$p = 0.980$, Pearson's r = −0.077

$p = 0.476$, Pearson's r = 0.013

Note: Analysis done for asthma patients only. Adjusted for Age, Gender, BMI and Smoking. pg/mL: picograms per milliliter; BMI: Body Mass Index; FVC: Forced Vital Capacity; FEV_1: Forced Expiratory Volume in 1 sec; PEF: Peak Expiratory Flow; 15-LO-1: 15-Lipoxygenase-1; PEBP1: Phosphatidylethanolamine-Binding Protein 1; AEC: Absolute Eosinophil Count.

Figure S3: A generalized linear model analysis was done in asthma patients to analyze the association between 15-LO-1/PEBP1 ratio and lung function parameters [percentage of predicted FVC (**A**), percentage of predicted FEV_1 (**B**), pre-FEV_1/FVC ratio (**C**), and pre-percent predicted PEF (**D**)], Eosinophil % (**E**), and AEC (**F**)].

Using GLM analysis in healthy controls, no significant association was observed between PEBP1 (pg/mL) levels and FVC pre-percent predicted, FEV_1 pre-percent predicted, pre-FEV_1/FVC ratio, PEF pre-percent predicted, Eosinophil % and AEC (Figure S4). Similarly, 15-LO-1 (pg/mL) levels (Figure S5) and 15-LO-1/PEBP1 ratio (Figure S6) showed no significant association with these parameters, except for eosinophil %, where a significant association was observed for both 15-LO-1 (Figure S5 E) and 15-LO-1/PEBP1 ratio (Figure S6E).

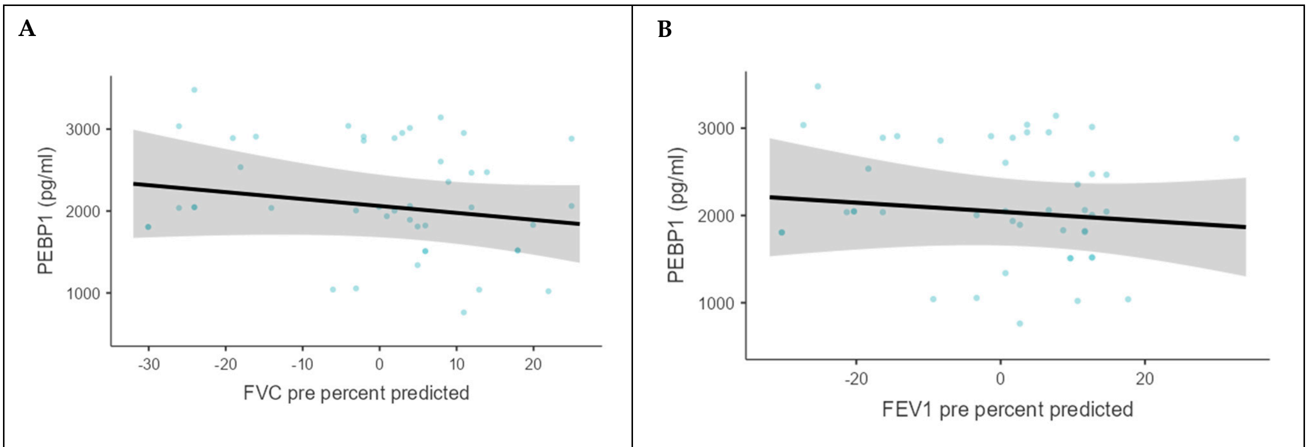

$p = 0.244$, Pearson's r = −0.225

$p = 0.486$, Pearson's r = −0.149

C

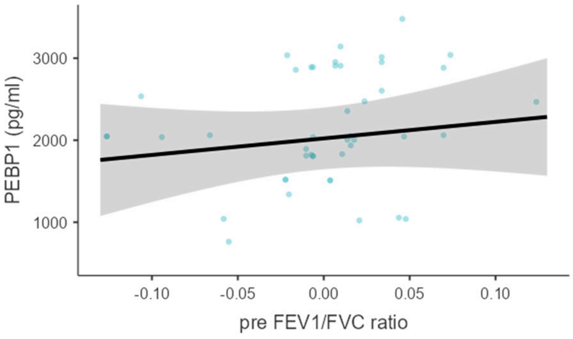

p = 0.376, Pearson's r = 0.224

D

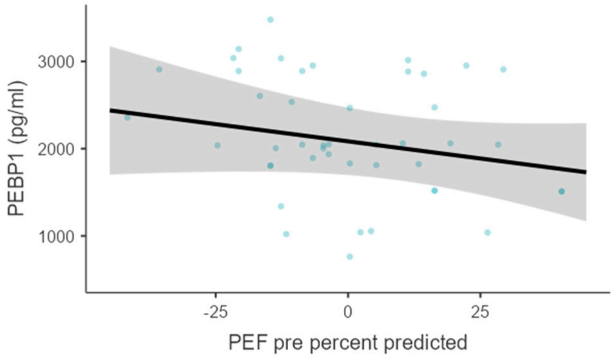

p = 0.183, Pearson's r = −0.271

E

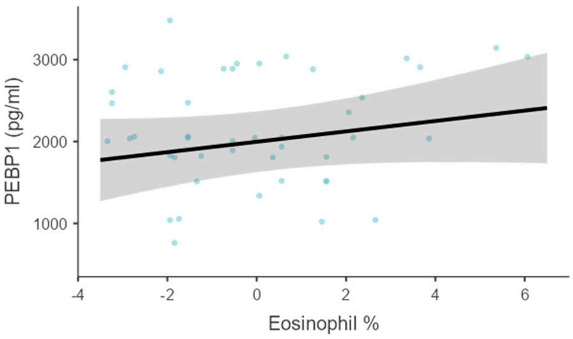

p = 0.164, Pearson's r = 0.158

F

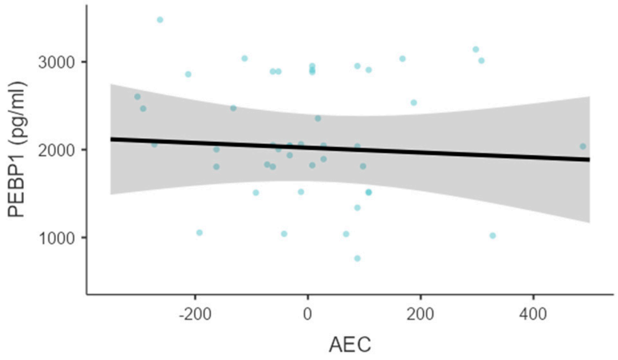

p = 0.676, Pearson's r = −0.078

Note: Analysis done for healthy controls only. Adjusted for Age, Gender, BMI and Smoking. pg/mL: picograms per milliliter; BMI: Body Mass Index; FVC: Forced Vital Capacity; FEV_1: Forced Expiratory Volume in 1 sec; PEF: Peak Expiratory Flow; PEBP1: Phosphatidylethanolamine-Binding Protein 1; AEC: Absolute Eosinophil Count.

Figure S4: A generalized linear model analysis was done in healthy controls to analyze the association between PEBP1 (pg/mL) and lung function parameters [percentage of predicted FVC (**A**), percentage of predicted FEV_1 (**B**), pre-FEV_1/FVC ratio (**C**), and pre-percent predicted PEF (**D**)], Eosinophil % (**E**), and AEC (**F**)].

A

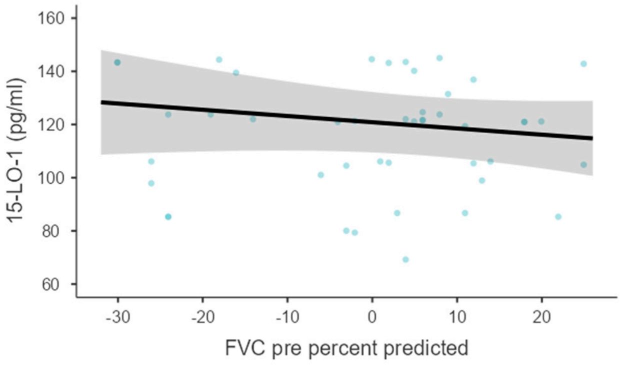

p = 0.278, Pearson's r = −0.029

B

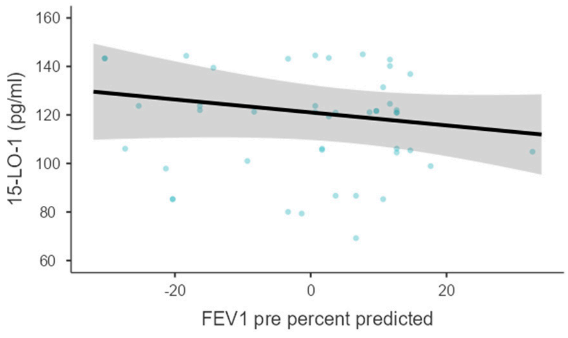

p = 0.223, Pearson's r = −0.077

C

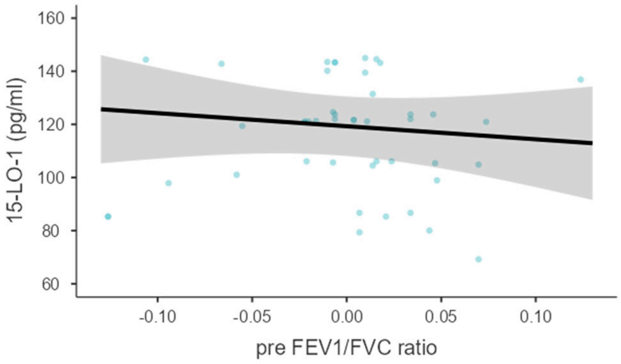

p = 0.467, Pearson's r = 0.004

D

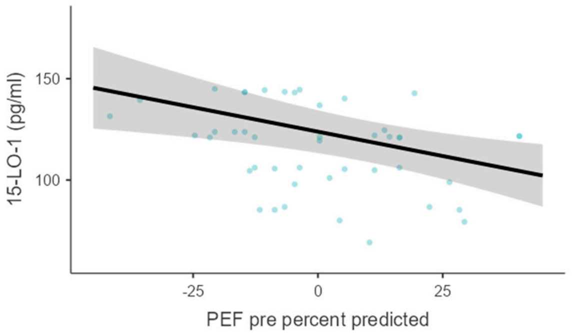

p = 0.004, Pearson's r = −0.324

E

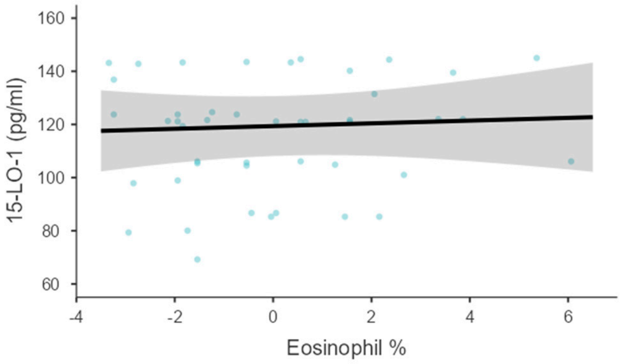

p = 0.707, Pearson's r = 0.109

F

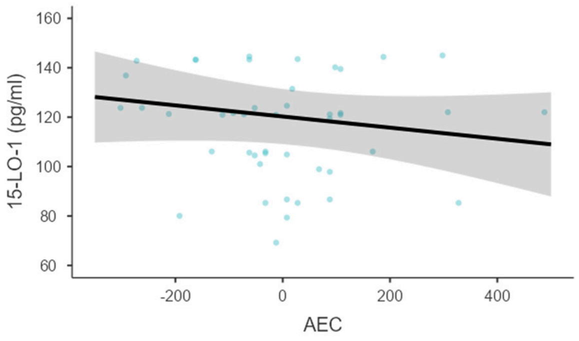

p = 0.243, Pearson's r = −0.083

Note: Analysis done for Healthy controls only. Adjusted for Age, Gender, BMI and Smoking. pg/mL: picograms per milliliter; BMI: Body Mass Index; FVC: Forced Vital Capacity; FEV_1: Forced Expiratory Volume in 1 sec; PEF: Peak Expiratory Flow; 15-LO-1: 15-Lipoxygenase-1; AEC: Absolute Eosinophil Count.

Figure S5: A generalized linear model analysis was done in asthma patients to analyze the association between 15-LO-1 (pg/mL) and lung function parameters [percentage of predicted FVC (**A**), percentage of predicted FEV_1 (**B**), pre-FEV_1/FVC ratio (**C**), and pre-percent predicted PEF (**D**)], Eosinophil % (**E**), and AEC (**F**)].

A

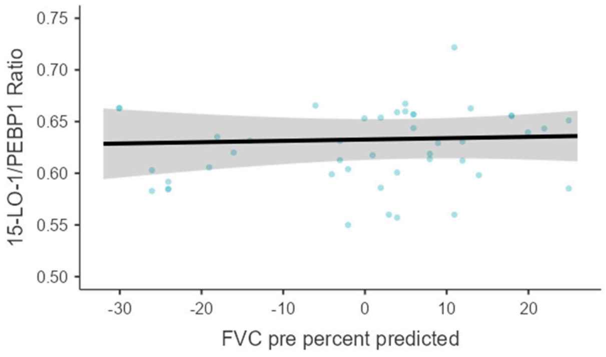

p = 0.729, Pearson's r=0.185

B

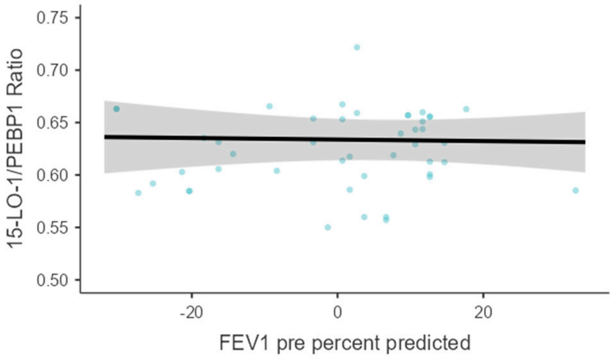

p = 0.846, Pearson's r = 0.079

C

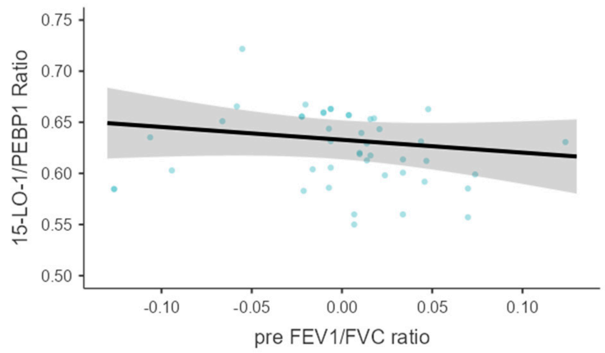

p = 0.277, Pearson's r = −0.156

D

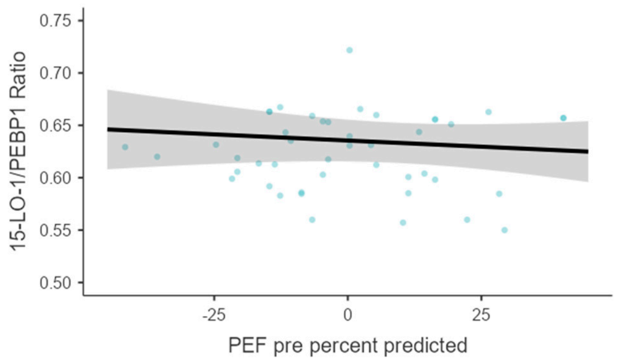

p = 0.439, Pearson's r = −0.008

E

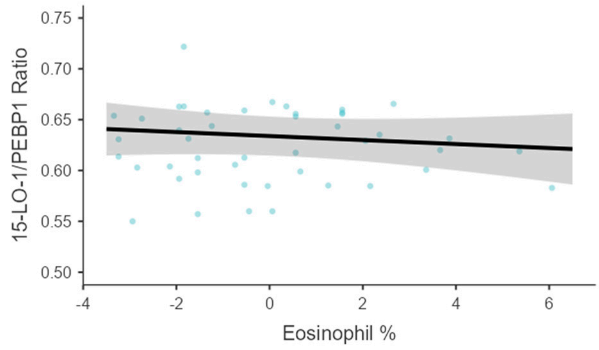

p = 0.401, Pearson's r = −0.033

F

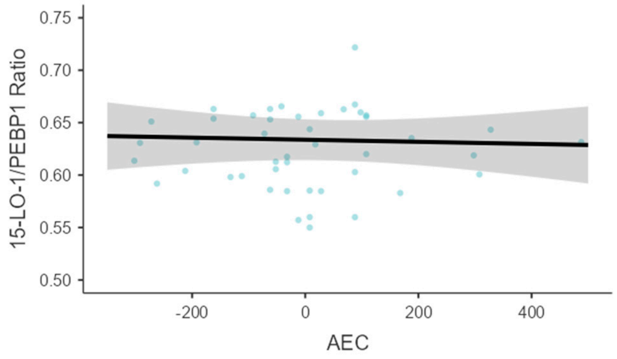

p = 0.765, Pearson's r = 0.034

Note: Analysis done for healthy controls only. Adjusted for Age, Gender, BMI and Smoking. pg/mL: picograms per milliliter; BMI: Body Mass Index; FVC: Forced Vital Capacity; FEV_1: Forced Expiratory Volume in 1 sec; PEF: Peak Expiratory Flow; 15-LO-1: 15-Lipoxygenase-1; PEBP1: Phosphatidylethanolamine-Binding Protein 1; AEC: Absolute Eosinophil Count.

Figure S6: A generalized linear model analysis was done in asthma patients to analyze the association between 15-LO-1/PEBP1 ratio and lung function parameters [percentage of predicted FVC (**A**), percentage of predicted FEV_1 (**B**), pre-FEV_1/FVC ratio (**C**), and pre-percent predicted PEF (**D**)], Eosinophil % (**E**), and AEC (**F**)].
